# The Yin & Yang of Mitochondrial Architecture - Interplay of MICOS and F_1_F_o_-ATP synthase in cristae formation

**DOI:** 10.15698/mic2017.08.583

**Published:** 2017-08-07

**Authors:** Heike Rampelt, Martin van der Laan

**Affiliations:** 1 Institute of Biochemistry and Molecular Biology, ZBMZ, Faculty of Medicine, University of Freiburg, 79104 Freiburg, Germany.; 2 Medical Biochemistry and Molecular Biology, Center for Molecular Signaling, PZMS, Saarland University, School of Medicine, 66421 Homburg, Germany.

**Keywords:** mitochondria, cristae, membrane architecture, contact site, MICOS, F_1_F_o_-ATP synthase, Mic10, respiration

## Abstract

Oxidative phosphorylation takes place at specialized compartments of the inner mitochondrial membrane, the cristae. The elaborate ultrastructure of cristae membranes enables efficient chemi-osmotic coupling of respiratory chain and F_1_F_o_-ATP synthase. Dynamic membrane remodeling allows mitochondria to adapt to changing physiological requirements. The mitochondrial contact site and cristae organizing system (MICOS) and the oligomeric ATP synthase have been known to govern distinct features of cristae architecture. A new study 1 on the crosstalk between these two machineries now sheds light on the mechanisms of cristae formation and maintenance.

Cristae membranes are connected to the planar inner boundary membrane (IBM) by a defined structure, the crista junction. This narrow and highly curved membrane domain is thought to enable the asymmetric distribution of proteins between the two major compartments of the inner mitochondrial membrane. Crista junctions require the conserved mitochondrial contact site and cristae organizing system (MICOS) for their formation and stability 2,3,4,5,6. At least six different genuine MICOS subunits form a large hetero-oligomeric complex that comprises the Mic60 subcomplex (including Mic60 and Mic19, in vertebrates additionally Mic25) and the Mic10 subcomplex (with Mic10, Mic12, Mic26 and Mic27) 7,8,9,10. The two core components Mic60 and Mic10 have non-redundant roles within MICOS and, accordingly, both are critical for the formation of stable crista junctions. Deletion of either of these subunits in the yeast *Saccharomyces cerevisiae* results in detachment of cristae membranes from the IBM and accumulation of lamellar membrane stacks within the mitochondrial matrix 2,3,4. Mic60 binds to several outer membrane protein complexes and is thus involved in the formation of membrane contact sites within mitochondria (reviewed in 11). Mic10 assembles into large membrane-bending oligomers 8,12, and the Mic10 subcomplex appears to crosstalk with the phospholipid environment (reviewed in 6).

Whereas MICOS is localized at crista junctions 2,13,14, the F_1_F_o_-ATP synthase mainly accumulates at the rims and edges of cristae membranes due to the angled conformation of its dimers 15,16. In fact, ATP synthase dimerization and oligomerization may drive cristae biogenesis by inducing membrane curvature. Thus, the two major cristae-shaping machineries are thought to localize at sites with distinct membrane curvature and regulate complementary aspects of cristae morphology. Both complexes were proposed to play differential roles in the biogenesis of lamellar cristae membranes that may originate from mitochondrial fusion events 17. However, evidence has been emerging that MICOS and ATP synthase also influence each other. Mic60 levels are negatively correlated with ATP synthase oligomerization. Mic60 overexpression induces formation of membrane junctions and branches 13. The mechanistic basis of this phenotype appears to be an intrinsic membrane remodeling activity of Mic60 that has been attributed to an amphipathic α-helix within the intermembrane space domain of the protein and that substantially contributes to MICOS function 18,19. In contrast, Mic60 deletion favors excessive ATP synthase oligomerization, and the accumulation of lamellar stacks of detached cristae in MICOS-deficient mitochondria requires ATP synthase 4,13. These findings indicate that an imbalance between the membrane-shaping activities of MICOS and ATP synthase contributes to aberrant cristae morphologies. However, the molecular basis of this coordination has remained enigmatic until recently.

In this issue of *Microbial Cell*, an elegant new study by Eydt *et al.* 1 investigates the poorly understood crosstalk between MICOS and F_1_F_o_-ATP synthase in determining cristae architecture. Initially, the authors establish that Mic27, a subunit of the Mic10 subcomplex, acts in an antagonistic manner to Mic60, both within MICOS and regarding effects on ATP synthase oligomerization. Ablation of Mic60, as shown previously, severely affects crista junction stability, cristae morphology, and respiratory growth, whereas the ultrastructure of Mic27-deficient mitochondria suggests a partial disruption of MICOS. Interestingly, the authors find that additional loss of Mic27 in Mic60-deficient cells ameliorates the strong phenotype of *MIC60* deletion. Mic27 was previously shown to support the formation of large Mic10 oligomers that are considered key structural elements of the holo-MICOS complex 10. In line with this view and earlier studies 2,3,4, Eydt *et al.* demonstrate via a sophisticated complexome profiling approach that MICOS stability is indeed strongly impaired in the absence of Mic27. Mic60 and Mic27 are components of distinct MICOS subcomplexes that both contribute to membrane bending at crista junctions 7,8,10,18,19. Thus, a possible interpretation for the synthetic positive interaction of *MIC60* and *MIC27* deletions is that unbalanced Mic10 oligomerization in the absence of Mic60 exacerbates the observed ultrastructural defects, which is partially prevented by the additional deletion of Mic27. However, alternative explanations are conceivable: The proposed phospholipid-binding properties of Mic27 (reviewed in 6) may contribute to the observed phenotypes. Reconstitution of purified Mic27 into proteoliposomes will be required to find out if Mic27 itself also possesses membrane-remodeling activities.

Close examination of the protein complex profiles in *mic27Δ* mutant mitochondria then led to a key observation of the new paper: Mic10 was the only MICOS component still present in high-molecular weight complexes in the absence of Mic27. The size of these complexes resembled that of F_1_F_o_-ATP synthase dimers. This exciting finding is in perfect agreement with a very recent study by Rampelt *et al.* 20, who used mainly biochemical approaches to demonstrate that a fraction of Mic10 associates with ATP synthase dimers independent of MICOS integrity. In both studies, no other MICOS components were found associated with detergent-solubilized ATP synthase complexes in considerable amounts using blue native PAGE 1,20. Moreover, Mic10 can be chemically crosslinked to the ATP synthase dimerization factor Su e (Atp21/Tim11), which explains the observed specificity of Mic10 binding to dimeric ATP synthase complexes 1,20. Crosslinking of Mic10 to Su e demonstrates the physical proximity of Mic10 and dimeric ATP synthase in intact membranes. The interaction is independent of Mic27 1 and also independent of Mic12 20, the subunit connecting the two MICOS subcomplexes 10. The latter findings underscore the notion that Mic10 association with ATP synthase does not require an intact MICOS complex. Mic10-ATP synthase interaction may support the oligomerization ATP synthase dimers into extended oligomeric rows, because upon Mic10 overexpression, its binding to the ATP synthase as well as oligomer stability are enhanced 20. However, the conundrum here is that Mic10 deletion also results in moderate oligomer stabilization 1,20, which may be an indirect consequence of massive membrane remodeling induced by MICOS disruption as seen with *mic60Δ* mutant mitochondria. Thus, the biophysical properties of diversely shaped mitochondrial inner membrane structures in different mutant cells appears to at least partially overlap and/or mask the regulatory functions exerted by direct protein-protein interactions. Further studies will be required to unravel the exact mutual relationship between these two parameters.

Another open question is where in the mitochondrial inner membrane the interaction of Mic10 and dimeric ATP synthase takes place, because due to their molecular architecture, Mic10 and ATP synthase oligomers favor distinctly curved membrane regions, the crista junctions and tips, respectively. One possibility is that at least a small fraction of dimeric ATP synthase is recruited to MICOS complexes at the tubular neck of crista junctions, a region that may accommodate both curvature orientations 1 (Fig. 1, right). In such a scenario, Mic27 that also has been crosslinked to Mic10 1,8 may provide a connecting function leading to a hypothetical binding chain of ATP synthase - Mic10 - Mic27 - remaining MICOS subunits 1. Alternatively, a subpopulation of Mic10 not associated with other MICOS components may associate with the ATP synthase dimers at cristae tips and rims 20 (Fig. 1, left). This model is supported by the observations that Mic10 can bind the ATP synthase independently of an intact MICOS, and that overexpressed Mic10, which does not associate with MICOS 8, shows an enhanced ATP synthase interaction 20.

**Figure 1:**
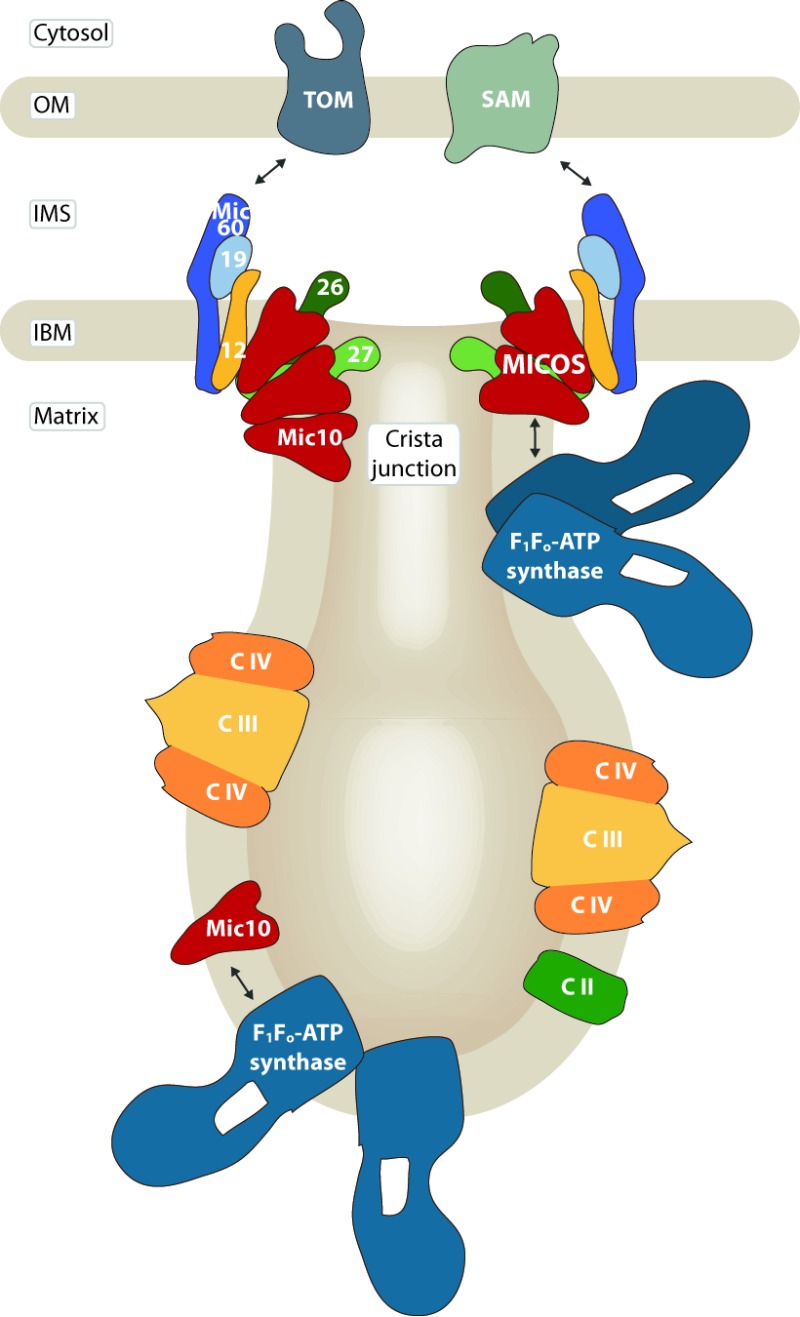
FIGURE 1: The mitochondrial contact site and cristae organizing system (MICOS) is required for the formation and stability of crista junctions of the inner mitochondrial membrane. Its core component Mic10 interacts with the F_1_F_o_-ATP synthase whose dimeric and oligomeric forms localize to strongly curved cristae tips and tubules. MICOS and the ATP synthase may associate via Mic10 at crista junctions (right), or a subpopulation of Mic10 may interact with the ATP synthase in cristae membranes (left). OM: outer membrane, IMS: intermembrane space, IBM: inner boundary membrane, TOM: translocase of the outer membrane, SAM: sorting and assembly machinery, C II / III / IV: complexes II, III and IV of the respiratory chain.

Taken together, exciting recent findings raise the possibility that the membrane-shaping activities of F_1_F_o_-ATP synthase and MICOS that control mitochondrial inner membrane architecture may be coordinated via the direct physical interaction of Mic10 with the ATP synthase. Cristae membranes are intensively remodeled in response to changes in physiological requirements, for example during metabolic adaptation 21,22. It is tempting to speculate that crosstalk between Mic10 and ATP synthase dimers and oligomers may be one point of attack for membrane remodeling forces and activities within mitochondria. Of note, the partner protein of Su e in promoting ATP synthase dimerization, Su g (Atp20), was shown to be phosphorylated. The phosphorylation state of Su g influences the assembly of dimeric ATP synthase 23,24. Such covalent modifications of small proteins connecting large protein complexes may be involved in regulatory circuits controlling mitochondrial inner membrane remodeling.
